# Clinical and radiographic changes in immediate implant placement versus delayed implant placement: An *in vivo* study

**DOI:** 10.6026/973206300213662

**Published:** 2025-10-31

**Authors:** Amit Siwach, Anil Sharma, Shruti Singh, Krati Singh, Omar Mukhtar, Ramesh Kumar

**Affiliations:** 1 Department of Prosthodontics and Crown & Bridge, Kalka Dental College, Meerut, Uttar Pradesh, India

**Keywords:** Bone density, CBCT, dental implants, delayed implant placement, immediate implant placement, peri-implant bone loss

## Abstract

The comparison between immediate and delayed dental implant placement is addressed in this study, with a focus on evaluating the clinical and radiological outcomes, including plaque scores, probing depths, soft tissue health, and bone density changes over six months. Therefore, it is of interest to compare immediate versus delayed dental implant placement, focusing on clinical and radiological outcomes. Key objectives included evaluating plaque scores, probing depths and soft tissue health and bone density changes over six months. A randomized trial with 20 implants (10 immediate, 10 delayed) was conducted, assessing clinical parameters at baseline, 3 and 6 months. Results showed that immediate implants initially had higher plaque scores and probing depths, but improved over time, while delayed implants demonstrated better bone density. Thus, both methods yielded favorable outcomes, with the choice of technique based on patient needs.

## Background:

Single tooth loss often causes aesthetic, psychological and functional issues, such as misalignment and non-physiologic occlusion [1]. Osseointegrated implants, particularly single-tooth implants, offer a reliable and natural alternative to fixed partial dentures by avoiding the preparation of adjacent teeth [2]. The introduction of osseointegration and the Branemark implant in 1982 marked a milestone in implant dentistry, improving bone transplant techniques and treatment efficiency [3]. Implants can be placed in healed sites (delayed) or fresh sockets (immediate). Immediate implants, placed post-extraction, reduce treatment time and preserve aesthetics but carry the risk of fibrous tissue growth instead of bone [4, 5]. The two-stage technique optimizes bone formation, while the one-stage technique simplifies the procedure [6]. Aesthetic success relies on proper soft tissue contours and a minimum of 5 mm between the contact point and crestal bone [7]. Keratinized mucosa of at least 2 mm is essential for gingival health, with thicker mucosa providing better resilience [8]. Recent studies suggest mucosal thickness affects crestal bone loss, with 3 mm needed for stability. This study aims to evaluate and compare clinical and radiological outcomes of immediate and delayed single-tooth implants in terms of gingiva width, mucosa thickness, papilla height, probing depth and bone loss [9, 10]. Therefore, it is of interest to describe the clinical and radiological differences in keratinized gingiva width, peri-implant mucosa thickness, papilla height, probing depth, soft tissue condition and bone loss between immediate and delayed single-tooth implant techniques.

## Methodology:

The present *in vivo* study was conducted on partially edentulous patients (males and females) aged 20-55 years, in the Department of Prosthodontics and Crown & Bridge, Kalka Dental College, Meerut, Uttar Pradesh. All treatment options were thoroughly discussed with the patients, including the relative advantages and disadvantages of implant treatment and patients were motivated accordingly. Patients were selected based on inclusion and exclusion criteria designed for the study. Those who were ready for the surgical placement of implants and subsequent follow-up appointments were included. Various materials and instruments used during the implant placement included patient drapes, local anesthetic agents, Betadine solution for gargling, normal saline, disposable gloves, syringes, cotton, suction tips, 3-0 sutures, extraction kits, diagnostic instruments (mouth mirrors, explorers, probes, tweezers, cheek retractors) and surgical kits (periosteal elevators, BP blade and handle, artery forceps, needle holders, tissue cutting scissors, suture cutting scissors). Also included were implant kits (Adin Advance), pilot drills, osteotomy drills, paralleling pins, depth probes, drill extenders, hex drives, implant drives, torque ratchets, two-piece implants and physiologic dispensers. Imaging was facilitated by OPG machines, IOPA machines, CBCT machines and the Marathon reduction handpiece, while Geistlich Bio-Oss bone grafts and curettes were also employed. A randomized, prospective clinical trial was conducted to evaluate clinical and radiological parameters of immediate and delayed implant placement. Fresh extraction sites were used with the immediate implant technique and healed sites with the delayed implant technique were followed. Ethical clearance was obtained from the institutional ethical board before the study's commencement.

A total of 10 sites in each group, across both genders and within the age limit of 20-55 years, were selected from the outpatient Department of Prosthodontics and Crown & Bridge based on the following selection criteria: patients were motivated for fixed implant support rehabilitation, had adequate bone width and height at the implant site, no signs of TMJ disorders, absence of systemic disease and good overall health and oral hygiene. Exclusion criteria included inability to undergo a minor oral surgical procedure, substance abuse history, unrealistic esthetic expectations and presence of vital anatomic structures near the implant site, insufficient bone quality or compromised health of the local site, inadequate mouth opening and insufficient vertical interarch space, among others. Diagnostic evaluations included medical history assessments, dental history and examinations and oral health assessments. Extraoral and intraoral examinations were performed to identify any abnormalities, such as lymphadenopathy or swellings. Blood pressure, pulse, temperature and respiration were measured and blood investigations were conducted to identify potential systemic diseases that could affect the surgical procedure and long-term implant success. Patients were assessed for bone quality and implant position through preoperative CBCT scans and a diagnostic wax-up and surgical stent were fabricated. Surgical procedures for both groups involved the administration of local anesthesia and the extraction of teeth, followed by implant placement in either fresh or healed sites, using appropriate drills and implant sizes based on anatomical site analysis. After surgery, antimicrobial prophylaxis (Amoxicillin 500 mg) was given and post-surgical analgesics (Paracetamol 500 mg + Aceclofenac 100 mg) were prescribed. Follow-up at 3 months included a second surgical procedure to mount healing abutments and after 4-6 weeks, final abutments were placed and prosthetic crowns were cemented. Clinical parameters, including the width and thickness of keratinized mucosa, plaque index, mucositis score, probing depth and soft tissue condition, were assessed at baseline, 3 months and 6 months post-surgery. Radiographic assessments involved CBCT imaging to evaluate bone density and peri-implant marginal bone loss. The data were subjected to statistical analysis to assess the differences between the immediate and delayed implant placement techniques.

## Results:

A total of 2 groups with 20 implants participated in this study, in which 10 implants were placed immediately after the tooth extraction and 10 implants were placed in healed extraction sockets. The implants were clinically and radiographically evaluated based on the implant placement. Table 1 (see PDF) shows the intergroup comparison of plaque scores between Group 1 (Immediate Implant Group) and Group 2 (Delayed Implant Group) at three time points: Baseline (1st Month), 3rd Month and 6th Month. At baseline, Group 1 had a higher mean plaque score of 1.313 compared to Group 2, which had a mean of 1.111. This difference was statistically significant with a p-value of 0.007, indicating that Group 1 had significantly higher plaque scores at the start of the study. At the 3rd month, Group 1 again showed a higher mean plaque score of 1.297, while Group 2 had a mean of 1.068. The p-value for this comparison was 0.045, which is statistically significant; reinforcing that Group1 had higher plaque scores than Group 2 at this stage as well. However, by the 6th month, the difference between the two groups was statistically significant. Group 1's mean score was 1.137, while Group 2's mean was 0.866. The p-value at this time point was 0.032, indicating that the difference between the groups was significant at the 6th month. Overall, Group 1 demonstrated significantly higher plaque scores than Group 2 at the 1st, 3rd and 6th months. Table 2 (see PDF) shows the intragroup comparisons of plaque scores in both the Immediate Implant (Group 1) and Delayed Implant (Group 2) groups, which revealed a progressive reduction in plaque scores over time. Group-1 Immediate Implant Group, Group-2 Delayed Implant Group At baseline (1st month), Group 1 had a mean plaque score of 1.313 ± 0.107, while Group 2 had a mean score of 1.111 ± 0.178. Over time, plaque scores decreased in both groups. By the 3rd month, Group 1's mean plaque score slightly reduced to 1.297 ± 0.309 and by the 6th month, it further declined to 1.137 ± 0.118. Similarly, Group 2 showed a gradual reduction in plaque scores, with a mean of 1.068 ± 0.203 at the 3rd month and 0.866 ± 0.214 at the 6th month. The intragroup change from baseline to 3rd Month to 6th Month was statistically significant in both groups (Figure 1 - see PDF). Table 3 (see PDF) shows the intergroup comparison of oral mucositis scores between the Immediate Implant Group (Group 1) and the Delayed Implant Group (Group 2), which revealed statistically significant differences at all-time points.

At baseline (1st month), Group 1 exhibited a higher mean mucositis score of 2.300 ± 0.483 compared to Group 2, which had a mean score of 0.900 ± 0.737. This difference was statistically significant (p = 0.001). Over time, mucositis scores decreased in both groups, but Group 1 consistently exhibited higher scores than Group 2. Group 1 had a mean score of 1.200 ± 0.421, while Group 2 had a lower mean score of 0.600 ± 0.516. This difference remained statistically significant (p = 0.011). By the 6th month, further improvement was observed, with Group 1 showing a mean mucositis score of 0.700 ± 0.483 and Group 2 showing a lower score of 0.200 ± 0.421. The difference between groups remained statistically significant (p = 0.024). These findings suggest that oral mucositis was more pronounced in the Immediate Implant Group at all-time points, but both groups showed a progressive reduction in scores over six months. Table 4 (see PDF) shows the intragroup comparison of oral mucositis scores over time in both the Immediate Implant Group (Group 1) and the Delayed Implant Group (Group 2), showing a significant reduction at each time interval. In Group 1, the baseline (1st month) mean mucositis score was 2.300 ± 0.483, which significantly decreased to 1.200 ± 0.421 by the 3rd month (p = 0.001, significant). Further improvement was observed by the 6th month, with the score reducing to 0.700 ± 0.483, showing another considerable decline compared to the 3rd month (p = 0.001, significant). Similarly, in Group 2, the baseline mean mucositis score was 0.900 ± 0.737, which significantly decreased to 0.600 ± 0.516 by the 3rd month (p = 0.001, significant). By the 6th month, the mucositis score further reduced to 0.200 ± 0.421, indicating a continued considerable improvement (p = 0.001, important). These findings indicate that both groups experienced a steady and statistically significant decline in oral mucositis scores over time, reflecting progressive healing and adaptation following implant placement. Group 1 (Immediate Implant Group) consistently exhibited a greater width of keratinized gingiva compared to Group 2 (Delayed Implant Group) at all-time points. At baseline, Group 1 showed a mean of 4.400 ± 1.135, significantly higher than Group 2's 3.300 ± 1.059 (p = 0.043). By the 3rd month, Group 1's mean width was 4.000 ± 1.247, whereas Group 2's mean was significantly lower at 2.800 ± 0.632 (p = 0.014). At the 6th month, Group 1 had a mean of 3.700 ± 1.251 and Group 2 showed a reduced value of 2.400 ± 0.875 (p = 0.012) (Table 5 - see PDF). Both groups showed a significant reduction in keratinized tissue width over the 6 months. Group 1 exhibited a decrease from 4.400 ± 1.135 at baseline to 3.700 ± 1.251 at the 6th month (p = 0.034). Group 2 showed a similar trend, decreasing from 3.300 ± 1.059 at baseline to 2.400 ± 0.875 at the 6th month (p = 0.031) (Table 6 - see PDF). Despite both groups showing a gradual increase in mucosal thickness, no significant differences were observed between Group 1 and Group 2 at baseline, 3rd month, or 6th month (Table 7 - see PDF). Both groups demonstrated a statistically significant increase in peri-implant mucosal thickness over time, indicating a positive soft tissue adaptation (Table 8 - see PDF).

## Discussion:

The study observed that the immediate implant group had higher plaque scores initially compared to the delayed implant group. However, these scores decreased significantly over time, converging with the delayed group by the 6th month. At baseline, the immediate implant group exhibited a mean plaque score of 1.313 ± 0.107, compared to 1.111 ± 0.178 in the delayed group (p = 0.007). By the 6th month, the immediate group's plaque score had reduced to 1.137 ± 0.118, while the delayed group's score was 0.866 ± 0.214 (p = 0.032). Similarly, Schropp *et al.* (2015) [11] observed that early placement of implants resulted in better soft tissue adaptation over time, consistent with the improvement in plaque scores seen in the present study. Initial probing depths and mucositis scores were higher in the immediate implant group but showed significant improvement over time. At baseline, the immediate group had a mucositis score of 2.300 ± 0.483, compared to 0.900 ± 0.737 in the delayed group (p = 0.001). By the 6th month, the immediate group's mucositis score reduced to 0.700 ± 0.483, while the delayed group's score was 0.200 ± 0.421 (p = 0.024). These findings support the conclusions of Chang *et al.* (1999) [12], who found that implant-supported single teeth exhibited initially higher probing depths and mucositis scores, but showed improvement over time. This suggests that despite early challenges, immediate implants can result in favorable soft tissue health comparable to delayed implants. The study demonstrated that the immediate implant group consistently maintained a wider keratinized gingiva compared to the delayed implant group. At baseline, the immediate group had a mean keratinized gingiva width of 4.400 ± 1.135, compared to 3.300 ± 1.059 in the delayed group (p = 0.043). By the 6th month, the immediate group's width reduced to 3.700 ± 1.251, while the delayed group's width was 2.400 ± 0.875 (p = 0.012). These results are consistent with Bouri *et al.* (2008) [13], who reported that a wider zone of keratinized mucosa is linked to better peri-implant health. The preservation of a wider keratinized gingiva in the immediate implant group may contribute to better long-term peri-implant stability and a reduced risk of peri-implant diseases. Both groups showed an increase in peri-implant mucosal thickness over time, with no significant differences between them. At baseline, the immediate group had a mean mucosal thickness of 2.700 ± 0.674, compared to 2.300 ± 0.823 in the delayed group (p = 0.250). By the 6th month, the immediate group's thickness increased to 3.200 ± 0.632, while the delayed group's thickness reached 3.000 ± 0.816 (p = 0.548). These findings are in line with Jung *et al.* (2022) [14], who highlighted the importance of peri-implant mucosal thickness in maintaining peri-implant health and preventing recession. Both implant placement techniques resulted in similar soft tissue adaptation, indicating that both approaches can achieve stable peri-implant mucosal thickness. The immediate implant group showed a significant reduction in bone density across all regions-buccal, apical and lingual-while the delayed implant group exhibited significant bone density increases. In the buccal region, the immediate group experienced a mean decrease of -27.60 ± 364.36, while the delayed group showed an increase of 114.50 ± 212.04 (p = 0.301). In the apical region, the immediate group showed a mean decrease of -56.00 ± 365.61, while the delayed group exhibited an increase of 208.50 ± 89.42 (p = 0.059). The lingual region saw a decline of -53.70 ± 244.67 in the immediate group, with a corresponding rise in 219.70 ± 199.35 in the delayed group (p = 0.397). These results are consistent with Patel *et al.* (2023) [15], who found that delayed implants generally show better bone density outcomes compared to immediate implants. The reduction in bone density in the immediate implant group may be attributed to the trauma associated with extraction and implant placement, potentially leading to bone resorption. However, the lack of significant differences in bone loss between the two groups at the 3rd and 6th months suggests that both methods can stabilize bone levels over time. There were no significant differences in bone loss between the immediate and delayed implant groups at the 3rd and 6th months. At the 3rd month, the immediate group had a mean mesial bone loss of 1.030 ± 0.427, while the delayed group had 1.225 ± 0.506 (p = 0.365). By the 6th month, the immediate group's mesial bone loss increased to 1.264 ± 0.429, while the delayed group showed 1.472 ± 0.394 (p = 0.274). These findings are in line with Arad *et al.* (2004) [16], who reported no significant differences in bone loss between immediate and delayed implants, despite the influence of implant timing on cervical bone loss. The results of this study carry significant clinical implications. First, the initial higher plaque scores and probing depths in the immediate implant group indicate that more intensive oral hygiene may be required during the early stages of healing. However, the improvement observed over time suggests that immediate implants can achieve soft tissue health outcomes similar to delayed implants. Second, the maintenance of a wider keratinized gingiva in the immediate implant group highlights the potential benefits of immediate implants in preserving peri-implant tissues. Finally, the findings suggest that although immediate implants can lead to initial bone resorption, both immediate and delayed implants can achieve stable bone levels over time, supporting the use of either technique based on the clinical situation. The studies by Kumar *et al.* (2023) [17] and Muthaiyan *et al.* (2025) [18] share several key similarities with the present study on immediate and delayed dental implant placement. Both these studies focus on comparing the clinical outcomes of immediate versus delayed implants, with an emphasis on parameters such as soft tissue health, bone density, and bone loss. Kumar *et al.* (2023) discussed the clinical outcomes, including soft tissue adaptation, which is similar to our evaluation of mucositis scores and keratinized gingiva width. Likewise, Muthaiyan *et al.* (2025) assess bone density changes, a key outcome also monitored in our study.

## Conclusion:

We show that while both the Immediate and Delayed Implant Groups showed significant changes in mucosal thickness and keratinized gingiva width, no significant differences in mucosal thickness were observed between the groups. Group 1, however, consistently exhibited greater keratinized gingiva width compared to Group 2 at all-time points. Thus, Bone density analysis indicated varying results between the two groups but did not show statistical significance across most regions.
